# Role of Genetic Polymorphisms in the Development of Complications in Patients with Implanted Left Ventricular Assist Devices: HeartWare, HeartMate II, and HeartMate 3

**DOI:** 10.3390/jcm12237235

**Published:** 2023-11-22

**Authors:** Madina R. Zhalbinova, Saule E. Rakhimova, Ulan A. Kozhamkulov, Gulbanu A. Akilzhanova, Assel A. Chinybayeva, Kenes R. Akilzhanov, Nurlan K. Shaimardanov, Anargul G. Kuanysheva, Joseph H. Lee, Ulykbek Y. Kairov, Makhabbat S. Bekbossynova, Ainur R. Akilzhanova

**Affiliations:** 1 National Laboratory Astana, Nazarbayev University, Astana 010000, Kazakhstan; madina.zhalbinova@nu.edu.kz (M.R.Z.); saule.rakhimova@nu.edu.kz (S.E.R.); ulan.kozhamkulov@nu.edu.kz (U.A.K.); ulykbek.kairov@nu.edu.kz (U.Y.K.); 2 Department of General Biology and Genomics, L. N. Gumilyov Eurasian National University, Astana 010000, Kazakhstan; 3 Department of Medicine, Semey Medical University, Pavlodar Branch, Pavlodar 140000, Kazakhstan; gulbanu.akilzhanova@smu.edu.kz (G.A.A.); kenes.akilzhanov@smu.edu.kz (K.R.A.); 4 Republic Diagnostic Center, CF “University Medical Center”, Astana 010000, Kazakhstan; assel.chinybayeva@umc.org.kz; 5 Department of Medicine, Semey Medical University, Semey 071400, Kazakhstan; nurlan.shaimardanov@smu.edu.kz (N.K.S.); anargul.kuanysheva@smu.edu.kz (A.G.K.); 6 Sergievsky Center, Taub Institute, Columbia University Medical Center, New York, NY 10032, USA; jhl2@cumc.columbia.edu; 7 National Research Cardiac Surgery Center, Astana 010000, Kazakhstan; makhabbat.bekbossynova@umc.org.kz; 8 Atomic Bomb Disease Institute, Nagasaki University, Nagasaki 852-8523, Japan

**Keywords:** genotype, polymorphism, heart failure, left ventricular assist device (LVAD), mechanical circulatory support (MCS)

## Abstract

Left ventricular assist device (LVAD) implantation is one of the mechanical circulatory support (MCS) treatments for advanced heart failure (HF) patients. MCS has emerged as a lifesaving therapy that improves patients’ quality of life. However, MCS remains limited by a paradoxical coagulopathy accompanied by thrombosis and bleeding. The mechanisms of MCS thrombosis are increasingly being defined, but MCS-related bleeding, which is related to shear-mediated alteration of platelet function, remains poorly understood. Complications might develop due to the high non-physiological shear stress in the device and as a consequence of individual variability in response to the antithrombotic therapy. Thromboelastography (TEG) and genotyping of gene polymorphisms that are involved in the coagulation cascade and in the metabolism of the antithrombotic therapy might be valuable sources of information for the reduction of complication development. The aim of the study was to identify genetic factors related to the development of device complications according to the implanted LVAD type. We compared the clinical and genetic data of HF patients (*n* = 98) with/without complications with three types of implanted devices: HeartWare HVAD (HW), HeartMate II (HMII), and HeartMate 3 (HM3). rs9923231 in *VKORC1* (95%CI −6.28–0.22, *p* = 0.04) and rs5918 in *ITGB3* genes (95%CI 0.003–4.36, *p* = 0.05) showed significant association with the TEG coagulation index parameter, which identified hyper- and hypo-coagulation states. The wild genotype of rs5918 in the *ITGB3* gene prevailed in patients implanted with HM3 devices, which developed fewer complications than with HMII (*p* = 0.04). Individual genetic information could be useful in the management of patients with HF and the implantation of MCS to reduce the development of complications.

## 1. Introduction

Mechanical circulatory support (MCS) has emerged as a lifesaving therapy for advanced heart failure (HF) patients, which improves patients’ quality of life. Left ventricular assist device (LVAD) implantation is one of the MCS treatments utilized as a bridge to transplantation (BTT) or destination therapy (DT) [1,2]. The utilization of LVADs in end-stage heart failure has doubled in the past ten years and is bound to continue to increase [1].

Since the first of these devices was implemented in 1994, the technology has changed tremendously, and so has the medical and surgical management of these patients [1]. The first generation of LVAD with pulsatile-flow technology had one-way valves and pumping chambers that often caused device breakdown and failure [3]. Currently, there are three types of the newer generation continuous-flow LVADs (CF-LVADs) that are implanted: HeartMate II (HMII) with an axial pump, HeartWare HVADs (HW), and HeartMate 3 (HM3) with a centrifugal pump [4]. Although dramatically lower adverse event rates have been achieved with the CF-LVADs as compared with older pulsatile-flow technology, thrombotic and bleeding complications remain unacceptably high in this population [5].

The treatment of end-stage HF with LVAD consistently improves patient quality of life and leads to decreased mortality levels [1,6,7]. However, despite these improvements, LVAD complications occur in 33% of patients waiting for heart transplantation [8,9,10,11].

Complications and adverse effects are the main reasons for the increased number of hospitalizations occurring within 90 days (more than 30%) and up to 12 months (more than 70%) after LVAD implantation [12]. Pump thrombosis and various post-LVAD bleeding events are the most frequent complications [1,13]. Pump thrombosis is one of the LVAD complications that requires pump exchange in HF patients [14]. The investigations identified various types of complications, which were compared between devices. For instance, bleeding and gastrointestinal bleeding events were found to be more prevalent in HMII devices than in HM3 devices [1,9,15,16]. And also, complications such as high risks of stroke, right heart failure, and sepsis were found to be associated with implanted HW devices in patients compared to HMII devices with axial pumps [16,17]. Complications occur due to platelet dysfunction, which is caused by the high non-physiological shear stress at the blade region of the device’s rotary. Shear stress by continuous flow pumps causes the degradation of high-molecular-weight multimers of von Willebrand factor, loss of the platelet glycoprotein receptors (GPIbα, GPVI, and GPIIb/IIIa), shedding, and damage, which affects hemostatic function [9,11,18,19]. For instance, the dysfunction of platelet receptors GPIIb/IIIa and GPIbα leads to bleeding and thrombosis events [11,20].

Antithrombotic therapy such as aspirin (antiplatelet) and warfarin (anticoagulant) is usually prescribed for HF patients to prevent pump thrombosis after device implantation, which has proven to be effective in the prevention of thromboembolic complications [21]. However, warfarin has a narrow therapeutic index and a wide variation in inter-individual dose requirements, which may contribute to bleeding and thrombotic complications because of over- and/or under-coagulation. Also, warfarin and aspirin dose prescription after LVAD implantation might be challenging because of the chronic underlying conditions that were treated with anticoagulants in the past. However, thrombosis and bleeding complications might occur due to the prescribed incorrect dosage of the drug [6,13].

Currently, genotype polymorphisms of *VKORC1* (vitamin K epoxide reductase complex 1) and *CYP2C9* (cytochrome P450 2C9) genes could prevent over- and under-coagulation of warfarin dosage, which can reduce complications. Growing lines of evidence suggest that genetic variants in these two genes are involved in the metabolism and action of warfarin and account for roughly 40% to 50% of the variability observed in warfarin dosing [11,13]. On the contrary, the process of aspirin metabolism occurs in two phases: deacetylation to salicylic acid after absorption. One of the major enzymes is UDP-glucuronosyltransferases (UGTs), which is involved in the second phase of aspirin metabolism [11]. The different genotypes of polymorphism rs2070959 in the *UGT1A6* gene are associated with decreased and increased enzyme activity, which results in faster or slower excretion of aspirin metabolites [11,22]. We have recently identified that mutant the GG genotype polymorphism of rs2070959 in the *UGT1A6* gene is associated with thrombosis/bleeding complications in HF patients with implanted LVAD (*p* = 0.03) [11]. The investigations show that gene polymorphisms play a necessary role in warfarin/aspirin metabolism, which could predict the amount of recommended dose to prevent LVAD complications [11,13]. Although warfarin and aspirin genotyping information can be incorporated into decision making for initial dosing, as recommended by the Food and Drug Administration, the clinical utility of this data in the CF-LVAD population has not been well studied.

On the other hand, thrombosis complications can be predicted using the method of thromboelastography (TEG) [23]—a viscoelastic hemostatic assay that measures the global viscoelastic properties of whole blood clot formation. TEG analysis evaluates the stage from platelet coagulation to lysis in a low shear stress state [23,24]. For coagulation analysis, TEG includes parameters starting from clot initiation (R-time) to increasing clot strength (*k*—clot kinetics, α—angle), to clot strength and stability (MA—maximal amplitude, G), and clot dissolution (LY30). The TEG coagulation index (CI) is one of the parameters that combines different TEG parameters with the identification of hyper- and hypo-coagulation states [23,24].

The investigations have concluded that complications could be predicted by the TEG method and eliminated by the prescription of antithrombotic/anticoagulant treatment according to the results of the genotype polymorphisms of the *VKORC1*, *CYP2C9*, and *UGT1A6* genes. Thus, our research aims to identify the genetic factors related to the development of device complications according to the implanted LVAD type. We hypothesize that genetic factors might influence the development of complications apart from LVAD’s shear stress, which could help predict the development of future complications during the pre/post-LVAD implantation period in HF patients.

## 2. Materials and Methods

### 2.1. Study Participants

The protocol was approved by the Ethics Committees at each center: at National Laboratory Astana, Nazarbayev University (No. 16 from 11 March 2015), and at the National Research Cardiac Surgery Center (NRCC), Astana, Kazakhstan (No. 16 from 24 April 2015). Written informed consent was received from all participants. The research was performed in accordance with the Declaration of Helsinki.

One hundred HF patients (age ≥ 18) with implanted continuous flow LVADs were recruited consecutively for this case series study during 2011–2016 at the NRCC. Two patients were excluded from the analysis as they were under 18 years old (9 and 16 years old). LVADs were implanted as BTT and DT according to the patient’s medical indications at their end-stage. HF patients had three years of follow-up from 2014 to 2017 with a median of 18 months. Patients (≥18 years old) had a diagnosis of ischemic cardiomyopathy (*n* = 44, ICM), dilated cardiomyopathy (*n* = 40, DCM), hypertrophic cardiomyopathy (*n* = 11, HCM), and valvular heart disease (*n* = 3, VHD). Patients were implanted with three types of continuous flow LVADs, such as HeartWare HVADs (HW) (HeartWare Inc., Framingham, MA, USA), HeartMate II (HMII) (Thoratec Corporation, Pleasanton, CA, USA), and HeartMate 3 (HM3) (St. Jude Medical, Huntingdon, Cambridgeshire, UK).

Clinical and epidemiological data were collected from the medical records of HF patients by cardiologists at NRCC (M.S.B). Baseline demographic parameters (age, gender, and ethnicity), anthropometry (height, weight, and body mass index (BMI)), LVAD type, systolic blood pressure (SBP), diastolic blood pressure (DBP), echocardiography, TEG, clinical biochemical parameters, and others were included.

According to the clinical protocol of the Ministry of Healthcare of the Republic of Kazakhstan, warfarin and aspirin anticoagulants were prescribed for the long term as the main therapy after LVAD implantation. Daily warfarin dose (2.99 ± 1.15 mg/day) was corrected to maintain the target international normalized ratio range (INR 2.25–3.25). The daily dose of aspirin was 100 mg/day. The baseline demographic characteristics of 98 HF patients are shown in Table 1.

TEG analysis was evaluated in HF patients once at the postoperative period (3–6 months) of LVAD implant during planned medical check-ups and during hospital re-admissions at NRCC.

Venous blood samples were collected into sterile vacutainers with K2EDTA for genetic analysis.

We conducted a retrospective study of HF patients and categorized them into three groups for comparative analysis according to the implanted type of LVADs: Group 1 (*n* = 18, HW), Group 2 (*n* = 34, HMII), and Group 3 (*n* = 46, HM3) (Table 1). Baseline demographic characteristics (Table 1), biochemical parameters (Table 2; Supplementary Table S1), and TEG parameters (Supplementary Table S2) were compared between LVAD types (HW, HMII, HM3).

Also, we divided HF patients into two groups for comparative analysis: Group 1, without complications (*n* = 74); Group 2 with complications (*n* = 24). A group of patients with complications (*n* = 24) had thrombosis and bleeding events after LVAD implantation. Consequently, patients with complications were classified into two subgroups for comparative analysis for more details: thrombosis (Group 2-1) and bleeding (Group 2-2).

We compared thromboelastography (TEG) parameters between three groups: without complications, with thrombosis, and with bleeding (Supplementary Table S3). TEG analysis was performed for patients (*n* = 53) who were able to visit NRCC for planned medical check-out and re-hospitalization after LVAD implantation.

The study included ninety-five healthy individuals as a control group without any cardiovascular diseases at the time of recruitment and in the family history. The healthy group was obtained for case–control study to perform genetic analysis for the identification the frequency of alleles and genotypes. The healthy group was described previously [11].

### 2.2. Selection of the Single-Nucleotide Polymorphisms

We selected twenty-one single-nucleotide polymorphisms (SNP) that are associated with cardiovascular events, the coagulation system, metabolism of warfarin and aspirin [11]. A list of SNPs and primers is summarized in Supplementary Table S4.

### 2.3. DNA Extraction and SNP Genotyping

Genomic DNA was isolated from whole venous blood samples (200 µL) using the PureLinkTM Genomic DNA Mini Kit (Invitrogen, Carlsbad, CA, USA). The purity and quantity of the DNA were checked and recorded using Nanodrop 2000 (ThermoScientific, Waltham, MA, USA). Purified DNA was stored in a −80 °C freezer until it was used for DNA genotyping. DNA samples were genotyped for all 21 selected SNPs by using real-time polymerase chain reaction (qPCR) with allele discrimination using TaqMan Real-Time PCR Assay on a 7900HT Fast Real-Time PCR System (Applied Biosystems, Waltham, MA, USA).

### 2.4. Statistical Analysis

The normality of the distribution of continuous variables was evaluated by using a Kolmogorov–Smirnov test (*p* > 0.05). Continuous variables were compared between two groups using Student’s *t*-test and the non-parametric Mann–Whitney U test. Moreover, continuous variables between the three devices were compared using one-way ANOVA and the non-parametric Kruskal–Wallis test. Continuous variables were reported as mean ± standard deviation (SD). Categorical variables were presented as frequencies and percentages, which were compared using the chi-square test or Fisher’s exact test. Sample size and power analysis were identified using an online calculator at https://clincalc.com (accessed on 5 September 2021) [11]. The sample size achieved 0.85 (85%) of power with an alpha value of 0.05. Each group should contain at least 16 HF patients.

Hardy–Weinberg equilibrium (HWE) for genetic deviation was assessed using the chi-square test or Fisher’s exact test. Genetic association analysis between SNPs and HF patients, healthy controls, and HF patients with/without complications were evaluated by using odds ratios (OR) with 95% confidence interval (CI) and *p*-value. Logistic regression analysis was performed using the web tool https://snpstats.net/ (accessed on 9 March 2022). Bonferroni-adjusted *p*-values were assessed for the association of SNPs between multiple comparisons of the healthy control group and HF patients with/without complications.

Pearson’s correlation test was performed to identify the relationship between measurable parameters and the result was expressed as rho value. Furthermore, we performed a multiple linear regression model to identify the relationship between TEG CI and basic demographic parameters, warfarin/aspirin dose, and the polymorphisms of rs8050894, rs9934438, rs9923231 in *VKORC1* gene, rs5918 in *ITGB3* gene, and rs2070959 in *UGT1A6*. Multinomial logistic regression analysis was performed to identify the relationship between LVAD types (HW, HMII, HM3) and genotype polymorphisms, which were statistically significant according to multiple linear regression analysis. All statistical analysis was performed in the SPSS program, version 23 (SPSS, Chicago, IL, USA).

## 3. Results

### 3.1. Clinical Characteristics of HF Patients with Three Types of Implanted LVAD Devices: HW, HMII, HM3

The baseline demographic characteristics of HF patients and comparative analysis between three LVAD types of HF patients are summarized in Table 1. HF patients with higher BMI was more prevalent in patients with implanted HMII device than with HW device (29.7 ± 4.65 vs. 24.6 ± 4.74, *p* = 0.001). The mean duration of LVAD support was 24.1 ± 15.8 months until the end of the project (outcome measurements) (until 2017). HF patients with implanted HM3 devices had less duration support due to the late implantation period, which was from 2015, whereas patients with HMII devices showed longer duration support since the 2011 implantation period, respectively (13.5 ± 7.56 vs. 38.3 ± 14.1, *p* = 0.001).

Seventy-one (72.4%) patients reached outcome measurement time, and among them, 41 (57.7) patients were implanted with HM3. On the other hand, twenty-seven (27.6%) of HF patients did not achieve the outcome, and most of them, *n* = 14 (51.9%), were implanted with an HMII device. In our patient cohort, thrombosis and infection events were more prevalent in HMII devices (61.5% and 46.2%) whereas most of the patients with HM3 devices (7.7% and 23.1%) had fewer complications (*p* < 0.05) (Table 1).

The biochemical parameters of pre- and post-LVAD implantation data were compared between three LVAD types (Table 2). Patients with implanted HW devices had significantly higher levels of D-Dimer, INR, and APTT levels at the pre-/post- LVAD implantation period (*p* < 0.05). On the other hand, patients with implanted HMII devices had significantly higher levels of hemoglobin, hematocrit, leukocytes, erythrocytes, LDH, and LVEF compared to the other two devices (*p* < 0.05). Creatinine and C-reactive protein levels were significantly higher in patients with HM3 devices. The rest of the biochemical parameters did not show a significant difference during the pre-/post-LVAD implantation period between the HMII, HM3, and HW devices (Supplementary Table S1).

TEG parameters were compared between three LVADs (Supplementary Table S2). Patients with implanted HMII devices had significantly lower levels of TEG, G (6.72 ± 3.27 d/s) compared to the other two devices (*p* = 0.02). TEG MA parameters were slightly significantly lower in patients with HMII devices (*p* = 0.06). On the other hand, TEG parameters did not reveal significant differences between the groups of patients without complications and with complications of thrombosis and bleeding (Supplementary Table S3). However, the parameter of TEG CI level was higher in the group of patients with thrombosis compared to the patients without complications, which showed slightly significant results (*p* = 0.07).

### 3.2. SNP Association with HF Patients

All HF patients (*n* = 98) and healthy control participants (*n* = 95) were genotyped for twenty-one selected SNPs. Out of twenty-one SNPs, five genotype polymorphisms rs8050894, rs9934438, rs9923231 in *VKORC1* gene, rs5918 in *ITGB3* gene, and rs2070959 in *UGT1A6* showed significant differences in the distributions of allelic and genotype frequencies between HF patients, healthy controls, and HF patients with/without complications (*p* < 0.05) (Supplementary Tables S5 and S6). Also, the two polymorphisms of rs8050894 in *VKORC1* and rs5918 in *ITGB3* genes showed significant association with HF patients according to the Bonferroni correction analysis (*p* < 0.001).

Furthermore, polymorphisms rs9934438, rs9923231 in the *VKORC1* gene, rs5918 in the *ITGB3* gene, and rs2070959 in *UGT1A6* were significantly associated with complications of HF patients according to the logistic regression analysis (*p* < 0.05) (Supplementary Table S7).

### 3.3. Correlation Analysis

**Figure 1:**
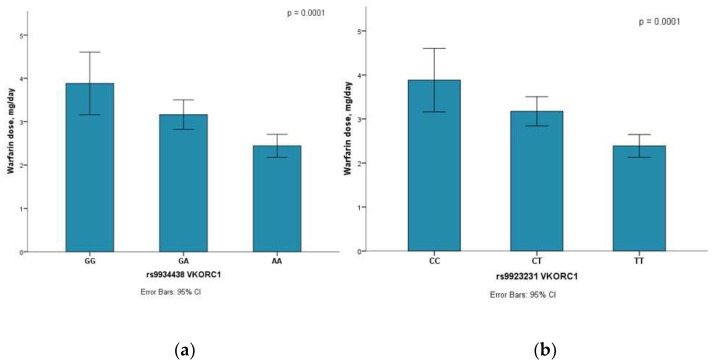
Warfarin dose among 3 genotypes of *VKORC1* gene. (**a**) Warfarin dose among three genotypes (GG, GA, AA) of rs9934438 in the *VKORC1* gene; (**b**) warfarin dose among three genotypes (CC, CT, TT) of rs9923231 in the *VKORC1* gene.

Pearson’s correlation test was performed to identify the relationship between age, BMI, device type, warfarin/aspirin dose, TEG CI, and five polymorphisms (Supplementary Table S8). There was a significant and negative correlation (rho = −0.433, *p* < 0.001) between warfarin dose level and polymorphism rs9934438 in the *VKORC1* gene. Polymorphism rs9923231 in the *VKORC1* gene also showed a significant and negative correlation (rho = −0.450, *p* < 0.001) with the warfarin dose. Furthermore, warfarin dose showed a significant difference between the genotypes of polymorphisms rs9934438 and rs9923231 in *VKORC1*. Thus, HF patients with wild-type (GG; CC) genotype polymorphisms of rs9934438 and rs9923231 in *VKORC1* were prescribed with higher warfarin dosage, whereas lower warfarin dosage was prescribed with the presence of mutant (AA; TT) genotype (Figure 1a,b).

### 3.4. Multiple Linear Regression Analysis

Multiple linear regression analysis was aimed to analyze the relationship between TEG CI and genetic polymorphism variables. The multiple linear regression analysis was adjusted for age, BMI, device type, warfarin/aspirin dosage, and genotype polymorphisms of rs8050894, rs9934438, rs9923231 in *VKORC1*, rs5918 in *ITGB3*, and rs2070959 in *UGT1A6* genes. The regression analysis demonstrated that warfarin dose (95%CI −1.66–0.40, *p* = 0.002), polymorphisms of rs9923231 in the *VKORC1* (95%CI −6.28–0.22, *p* = 0.04) and rs5918 in the *ITGB3* (95%CI 0.003–4.36, *p* = 0.05) genes are statistically significant independent predictors of TEG CI (Table 3).

### 3.5. Multinomial Logistic Regression Analysis

Multinomial logistic regression analysis was aimed to identify the relationship between LVAD type and the parameters of age, BMI, warfarin dose, and the genotype polymorphisms of rs9923231 in *VKORC1* and rs5918 in *ITGB3* genes (Table 4). The regression analysis demonstrated that higher BMI was more prevalent in HMII devices than in the reference device HM3 (OR 1.15, 95% CI 1.02–1.29, *p* = 0.02). On the other hand, between the two polymorphisms, rs5918 in *ITGB3* with TT genotype was more prevalent for patients with the HM3 device (OR 0.33, 95% CI 0.12–0.94, *p* = 0.04).

## 4. Discussion

This research aimed to investigate the genetic predictors of complication development in HF patients apart from the shear stress of LVAD according to the implanted device type. In this investigation, our analysis revealed two genotype polymorphisms, rs9923231 in the *VKORC1* and rs5918 in the *ITGB3*, that were significantly associated with HF patients with implanted LVAD types (*p* < 0.05).

Previously, we performed a comparative analysis between the groups of HF patients without complications and those with complications after LVAD implantation. We showed that the genotype polymorphisms of rs9934438 and rs9923231 in the *VKORC1* gene, rs5918 in the *ITGB3* gene, and rs2070959 in the *UGT1A6* were significantly associated with complications (thrombosis and bleeding) in HF patients (*p* < 0.05) [11]. Furthermore, in the present research, we studied the impact of LVAD devices (HW, HMII, and HM3) on the changes in biochemical parameters and their influence on the development of complications (thrombosis/bleeding) in HF patients. This study showed the influence of the LVAD device and genetic polymorphisms on the development of complications in HF patients (*p* < 0.05).

Mehra et al. (2019), in their final report, performed a comparison between both HM3 and HMII devices in the Multicenter Study of MagLev Technology in Patients Undergoing Mechanical Circulatory Support Therapy with HeartMate 3 (MOMENTUM 3). The results of the MOMENTUM 3 trial showed that the HM3 device was superior to the HM2 device in terms of the survival of HF patients. Complications such as bleeding events, stroke, and pump replacement were often observed with HMII devices [9]. In our research, we identified that thrombosis complications and changes in biochemical parameters were more prevalent in HF patients with implanted HMII devices than in patients with HM3 devices (*p* < 0.05). The investigations revealed that a higher level of lactase dehydrogenase (LDH) is associated with thrombosis events in HF patients [6,9,14]. Also, according to the MOMENTUM 3 trial, higher LDH levels were more prevalent in patients with HMII devices than in those with HM3 devices [9]. Consequently, our research also showed a significantly higher level of LDH in the HMII device rather than in the HM3 device after LVAD implantation (351.5 ± 92.1 vs. 251,1 ± 88.0, *p* = 0.002). And, our analysis showed that the HMII device was significantly associated with a higher level of hemoglobin (134.2 ± 12.6 vs. 127.2 ± 18.3, *p* = 0.005) and hematocrit (39.5 ± 3.85 vs. 36.5 ± 5.80, *p* = 0.02) compared with HM3 device. Moreover, this analysis showed that higher levels of hemoglobin and hematocrit could be predictors of thrombosis events in HF patients with HMII devices, as thrombosis complications were more prevalent in eight HF patients (61.5%) with HMII devices (Table 1). Previous investigations showed that bleeding complications were associated with lower levels of hemoglobin and hematocrit, which could be an obvious predictor of the bleeding events during the pre-/post-LVAD implantation period (*p* < 0.05) [11,25]. According to the results of the investigations, it is known that device complications are much less frequent with HM3 than with HM2, which is caused by the device itself (shear stress) and due to incorrect anticoagulant dosages [9,14]. On the other hand, genotype polymorphisms could be one of the additional factors that may influence complication development. For instance, the genotype polymorphisms of the *VKORC1* and *CYP2C9* genes that are associated with warfarin sensitivity are studied in HF patients with implanted devices [13]. However, other genotype polymorphisms that are associated with coagulation factors, metabolism of anticoagulant/antiplatelet therapy, and cardiovascular events are not well studied, especially in HF patients with implanted mechanical circulatory support devices [9,11,13]. Our research performed a study on genotype polymorphisms as they might be one of the additional factors in the development of complications, which may reduce complication risks in HF patients with implanted LVAD.

According to the investigations of Xia et al. [23], suspected thrombosis complications in HMII devices could be predicted by the parameter of TEG CI. On the contrary, an investigation by Piche et al. [26] revealed that there is no association between the development of LVAD thrombosis and TEG MA. Consequently, in our investigation, we preferred to analyze the TEG CI parameter as it describes the overall coagulation status (hypercoagulation/hypocoagulation) of HF patients [23,24].

In the present study, we aimed to identify the influence of the genotype polymorphisms of rs9923231 and rs9934438 in *VKORC1,* rs5918 in *ITGB3,* and rs2070959 in *UGT1A6* genes as additional factors of complication development, which were not performed before in CF-LVAD patients. No research analyzed the relationship between the TEG CI and SNPs in HF patients with implanted LVAD [23,24,26,27]. Our analysis showed that warfarin dose (95%CI −1.66–0.40, *p* = 0.002), polymorphisms of rs9923231 in the *VKORC1* (95%CI −6.28–0.22, *p* = 0.04), and rs5918 in the *ITGB3* (95%CI 0.003–4.36, *p* = 0.05) are correlated with the TEG CI parameter.

We found that warfarin and TEG CI parameters showed a negative correlation (rho = −0.386, *p =* 0.004), which showed lower warfarin dosing at positive TEG CI value according to the Pearson correlation analysis (Supplementary Table S8). These negative correlation results were achieved because HF patients were already receiving long-term warfarin treatment during TEG analysis. TEG analysis should be performed before the prescription of warfarin dose, which might help to identify the correct dose. However, investigations reveal that TEG is not an optimal tool to evaluate the warfarin effect compared with INR measurements [28,29].

*VKORC1* is one of the genes involved in warfarin metabolism and dose effects [13,30]. The genotype polymorphism of rs9923231 in the *VKORC1* gene showed an influence on the TEG CI parameter as it could predict warfarin dose according to the genotype results in individual patients. Warfarin dose by the genotype polymorphism of rs9923231 in the *VKORC1* helps to prevent the hypercoagulation and hypocoagulation of the drug during treatment. Studies by Topkara et al. (2016) and Awad et al. (2015) identified that the dose of warfarin differs between genotype polymorphism of rs9923231 in the *VKORC1* gene in HF patients with an LVAD device [13,30]. Consequently, the investigations revealed that HF patients were prescribed an increased dose of warfarin in the presence of the wild-type genotype. On the other hand, in the presence of the mutant genotype, HF patients were prescribed a reduced dose of warfarin. Our research also revealed that HF patients with the wild-type CC genotype were prescribed a higher warfarin dose, whereas those with mutant the TT genotype of the polymorphism of rs9923231 in the *VKORC1* gene were prescribed a lower dose (Figure 1b) [11,13].

The high non-physiological shear stress at the blade region of the LVAD’s rotary is one of the main factors that ruins normal hemostatic function by causing platelet dysfunctions [31]. Glycoprotein receptor GPIIb/IIIa is one of the main protein complexes that is abundantly expressed on the surface of the platelet membrane with about 40,000–80,000 copies on the platelet. It is a complex of membrane proteins that consists of two subunits, GPIIb and GPIIIa, which are formed via calcium-dependent association [32]. Consequently, in our research, we investigated the polymorphism of rs5918 in the *ITGB3* gene, which encoded one of the platelet receptors GPIIIa, to identify the influence of the genetically inherited platelet dysfunction on the development of LVAD complications apart from the effect of the non-physiological shear stress [19,33]. The investigations revealed that the mutant genotype of the rs5918 polymorphism in the *ITGB3* gene has a risk of thrombosis formation with increased levels of platelet activation, which is common for coronary artery diseases, stroke, and myocardial infarction [33,34]. In an earlier study, one of the platelet glycoprotein receptors was identified in the first generation of mechanical circulatory support devices. Potapov E.V. et al. (2004) found that the genetic polymorphism of platelet glycoprotein receptors may contribute to the development of complications in implanted devices [35]. According to the research results, the wild-type genotype showed a significant association with bleeding, whereas the heterozygote genotype with thromboembolic showed complications in patients with implanted devices. Consequently, in our study, the distribution analysis of the genotype polymorphism of rs5918 in the *ITGB3* gene between the thrombosis and bleeding complications showed that the TC genotype was significantly higher in HF patients with thrombosis complications than in patients without thrombosis (84.6 vs. 36.4%, *p* = 0.043) (Supplementary Table S9). On the other hand, the TC genotype of rs5918 polymorphism in the *ITGB3* gene was significantly higher in HF patients with an absence of bleeding complications than in patients with bleeding events (90.0 vs. 42.9%, *p* = 0.022). The distribution analysis showed that the identification of the TC genotype polymorphism of rs5918 in the *ITGB3* gene may reduce the risk of complication development in HF patients with implanted LVAD. The polymorphism of rs5918 in the *ITGB3* gene also showed a relationship with the TEG CI parameter as it characterizes blood coagulation status (hypercoagulation/hypocoagulation) according to the multiple linear regression analysis (*p* = 0.05). Furthermore, the polymorphism of rs5918 in the *ITGB3* gene showed significance according to the multinomial logistic regression analysis results [OR (95% CI): (0.33 (0.12–0.94), *p* = 0.04)]. The TT wild-type genotype of rs5918 polymorphism in the *ITGB3* gene was more prevalent in HF patients with the HM3 (60.9%) device than in those with the HMII (35.3%) device (Table 4; Supplementary Table S10).

Moreover, we searched for the distributions of the genotype polymorphisms of rs8050894, rs9923231, rs9934438 in *VKORC1,* rs5918 in *ITGB3,* and rs2070959 in *UGT1A6* genes between HW, HMII, and HM3 devices in more detail to reveal genotype significance in specific device types (Supplementary Table S10). Consequently, the frequency of genotype distribution between LVAD devices showed that the TC genotype of rs5918 polymorphism in the *ITGB3* gene was higher in HF patients with HMII devices than in those with HM3 devices (44.1% vs. 28.3%, *p* = 0.21) (Supplementary Table S10). The TC genotype of rs5918 polymorphisms in the *ITGB3* gene showed a significant association with LVAD complications in HF patients (OR (95% CI): 5.37 (1.79–16.16), *p* = 0.0056) (Supplementary Table S7). And, thrombosis complications occurred more in HF patients with HMII devices than in those with HM3 devices (61.5% vs. 7.7%, *p* = 0.005) (Table 1). Our research results identified that, indeed, the HMII device causes the development of thrombosis complications with itself, and the presence of the genetically inherited platelet’s glycoprotein receptor dysfunction may also cause changes to the normal hemostatic function followed by higher risks to the development of complications. Apart from LVAD’s non-physiological shear stress, the genotype polymorphism of rs5918 in the *ITGB3* gene is one of the additional factors that may enhance the risks of complication development. In future research, our study will use next-generation sequencing technologies to perform whole genome and exome sequencing of HF patients with LVADs as it may help to correct HF patients’ anticoagulant/antiplatelet therapies, reduce risks of complication development and mortality rate, and improve patient’s quality of life.

## 5. Conclusions

Comparative analysis between HW, HMII, and HM3 devices showed their influence on the development of the complications, which are followed by changes in the biochemical parameters. Our observation identified that monitoring of the biochemical parameters could be used in the detection of post-LVAD complications. Among the three devices, changes in biochemical parameters were more prevalent in HF patients with HMII devices, who were associated with thrombosis complications.

We also identified that the dysfunction of platelet receptors is caused not only by the shear stress of the LVAD device but it could also be inherited genetically. The polymorphism of rs5918 in the *ITGB3* gene was found to be associated with implanted HMII devices in HF patients and with thrombosis complications. On the other hand, the polymorphism of rs9923231 in the *VKORC1* gene might prevent over- and under-coagulation due to the prediction of the recommended warfarin dose. Genomic data would help to identify the dosage of anticoagulant treatment, which may reduce thrombosis/bleeding complications in HF patients with implanted LVAD. The genotype polymorphisms of the *ITGB3* and *VKORC1* genes are one of the additional factors of complication development in HF patients apart from the non-physiological shear stress of LVAD devices. This individual genetic treatment approach may improve clinical outcomes with reduced LVAD complications in HF patients, with fewer re-hospitalization cases, which will be economically profitable for the healthcare system.

## 6. Limitations

Our research involved 98 HF patients with implanted LVADs, which was not enough to perform more powerful statistical analyses between the HF groups. Genotyping was performed only for the main genotype polymorphisms (21 SNPs) associated with cardiovascular events, coagulation factors, and metabolism of anticoagulant/antiplatelet therapy. The investigations of additional genotype polymorphisms would allow the identification of more associations with complication development in HF patients. The TEG data for blood coagulation analysis were not available monthly due to the patients’ death or because they went to their hometowns after the LVAD implantation.

## Figures and Tables

**Table 1:** Baseline demographic characteristics of HF patients and comparison between HeartWare HVAD, HeartMate II, and HeartMate 3.

Characteristic	HF Patients, *n* = 98	HW (*n* = 18)	HMII (*n* = 34)	HM3 (*n* = 46)	*p* Value
Age (years)	52.7 ± 11.0	53.1 ±11.7	53.3 ± 10.9	52.0 ± 10.9	0.87
Gender					
Male	92 (93.9)	16 (17.4)	31 (33.7)	45 (48.9)	0.23
Female	6 (6.1)	2 (33.3)	3 (50.0)	1 (16.7)	
Ethnicity					
Asian	77 (78.6)	17 (22.1)	25 (32.5)	35 (45.5)	0.20
Caucasian	21 (21.4)	1 (4.8)	9 (42.9)	11 (52.4)	
Body weight (kg)	79.8 ± 13.9	70.7 ± 12.9	85.9 ± 15.3	78.9 ± 11.1	0.001 *
Height (cm)	169.8 ± 6.36	169.4 ± 5.1	169.9 ± 7.96	169.8 ± 5.56	0.97
BMI (kg/m)	27.7 ± 4.5	24.6 ± 4.74	29.7 ± 4.65	27.3 ± 3.53	0.001 **
SBP	104.8 ± 15.5	105.9 ± 19.4	106.2 ± 16.0	103.3 ± 13.5	0.51
DBP	71.2 ± 10.3	71.2 ± 10.8	71.2 ± 10.4	71.3 ± 10.3	0.98
History of smoking					
Smokers	58 (59.2)	10 (17.2)	17 (29.3)	31 (53.4)	0.28
Non-smokers	40 (40.8)	8 (20.0)	17 (42.5)	15 (37.5)	
Diagnosis					
ICM	44 (44.9)	9 (20.5)	16 (36.4)	19 (43.2)	0.78
DCM	40 (40.8)	7 (17.5)	15 (37.5)	18 (45.0)	
HCM	11 (11.2)	1 (9.1)	3 (27.3)	7 (63.6)	
VHD	3 (3.1)	1 (33.3)	0	2 (66.7)	
NYHA (before 14 days)					
I	1 (1.0)	0	1 (100)	0	0.48
II	1 (1.0)	0	1 (100)	0	
III	2 (2.0)	0	1 (50.0)	1 (50.0)	
IV	26 (26.5)	6 (23.1)	9 (34.6)	11 (42.3)	
IIIA	34 (34.7)	8 (23.5)	13 (38.2)	13 (38.2)	
IIIB	34 (34.7)	4 (11.8)	9 (26.5)	21 (61.8)	
HF type					
HFrEF	97 (99.0)	18 (18.6)	33 (34.0)	46 (47.4)	0.53
HFmrEF	1 (1.0)	0	1 (100.0)	0	
INR					
Basic INR	1.21 ± 0.36	1.33 ± 0.46	1.16 ± 0.21	1.20 ± 0.40	0.35
Target INR	2.39 ± 0.26	2.83 ± 0.26	2.30 ± 0.12	2.27 ± 0.12	0.001 **
Device strategy					
BTT	10 (10.2)	0	7 (70.0)	3 (30.0)	0.05 *
DT	88 (89.8)	18 (20.5)	27 (30.7)	43 (48.9)	
Warfarin dose (mg/day)	2.99 ± 1.15	2.99 ± 1.42	2.82 ± 1.02	3.11 ± 1.13	0.21
Duration of LVAD support till outcome, from 2011 until 2016, *n* = 36 (in months)	29.6 ± 17.3	26.0 ± 10.2	38.6 ± 16.0	10.9 ± 6.73	0.001 *
Duration of LVAD support till outcome in all HF patients, *n* = 98 (in months)	24.1 ± 15.8	24.4 ±13.4	38.3 ± 14.1	13.5 ± 7.56	0.001 **
Patients’ achieved outcomes till 2017					
Survived	71 (72.4)	10 (14.1)	20 (28.2)	41 (57.7)	0.001 *
Not-survived	27 (27.6)	8 (29.6)	14 (51.9)	5 (18.5)	
Thrombosis					
Yes	13 (13.3)	4 (30.8)	8 (61.5)	1 (7.7)	0.005 *
No	85 (86.7)	14 (16.5)	26 (30.6)	45 (52.9)	
Bleeding					
Yes	14 (14.3)	4 (28.6)	6 (42.9)	4 (28.6)	0.27
No	84 (85.7)	14 (16.7)	28 (33.3)	42 (50.0)	
Infections					
Yes	39 (39.8)	12 (30.8)	18 (46.2)	9 (23.1)	0.0001 *
No	59 (60.2)	6 (10.2)	16 (27.1)	37 (62.7)	
Stroke					
No Stroke	78 (79.6)	12 (15.4)	25 (32.1)	41 (52.6)	0.18
Hemorrhagic stroke	8 (8.2)	2 (25.0)	4 (50.0)	2 (25.0)	
Ischemic stroke	12 (12.2)	4 (33.3)	5 (41.7)	3 (25.0)	
Myocardial infarction					
Yes	44 (44.9)	9 (20.5)	16 (36.4)	19 (43.2)	0.77
No	54 (55.1)	9 (16.7)	18 (33.3)	27 (50.0)	

Continuous variables are presented as mean ± SD, and categorical variables as *n* (%). HF patients, heart failure patients; significant *p*-value with one asterisk (*) from one-way ANOVA; significant *p*-value with double asterisks (**) from Kruskal–Wallis test; the significant *p*-value (*p* < 0.05) is labeled in bold; “-”, not available parameters; HW, HeartWare HVAD; HMII, HeartMate II; HM3, HeartMate 3; BMI, body mass index; SBP, systolic blood pressure; DBP, diastolic blood pressure; ICM, ischemic cardiomyopathy; DCM, dilated cardiomyopathy; HCM, hypertrophic cardiomyopathy; VHD, valvular heart disease; NYHA, New York Heart Association; HFrEF, heart failure reduced ejection fraction; HFmrEF, heart failure mid-range ejection fraction; INR, International normalized ratio; BTT, bridge-to-transplantation; DT, destination therapy.

**Table 2:** Comparative analysis of biochemical parameters between HeartWare HVAD, HeartMate II, and HeartMate 3 devices.

Study Groups	Parameters	Before 14 Days	*p* Value	After 3–6 Months	*p* Value	After 12–18 Months	*p* Value
HW	D—Dimer, mcg/mL	1.34 ± 1.23	0.30	2.67 ± 3.20	0.28	1.38 ± 0.63	0.01 **
HMII		1.32 ± 1.85		0.98 ± 0.49		0.65 ± 0.30	
HM3		1.08 ± 1.74		1.97 ± 1.13		0.49 ± 0.26	
HW	Hemoglobin, g/L	132.8 ± 19.3	0.39	111.9 ± 23.0	0.005 **	102.9 ± 22.4	0.001 *
HMII		138.9 ± 22.2		134.2 ± 12.6		136.2 ± 13.8	
HM3		140.6 ± 17.0		127.2 ± 18.3		106.1 ± 18.5	
HW	Hematocrit, %	39.6 ± 5.14	0.55	34.2 ± 6.66	0.02 **	31.5 ± 6.54	0.001 *
HMII		40.9 ± 8.22		39.5 ± 3.85		40.1 ± 4.50	
HM3		41.7 ± 6.08		36.5 ± 5.80		32.0 ± 5.50	
HW	Leukocytes, ×10^9^/L	5.99 ± 1.10	0.25	5.98 ± 1.28	0.04 *	7.08 ± 1.93	0.92
HMII		6.44 ± 1.46		7.47 ± 1.49		6.94 ± 1.74	
HM3		6.68 ± 1.54		7.14 ± 1.94		6.73 ± 1.68	
HW	Erythrocytes, ×10^12^/L	4.89 ± 0.54	0.93	4.19 ± 0.36	0.03 *	3.93 ± 0.96	0.03 **
HMII		4.98 ± 0.76		4.69 ± 0.65		4.72 ± 0.49	
HM3		4.97 ± 0.63		4.22 ± 0.72		3.73 ± 0.83	
HW	INR	1.35 ± 0.49	0.35	2.76 ± 0.87	0.15	2.90 ± 1.04	0.03 *
HMII		1.29 ± 0.52		2.30 ± 0.77		2.21 ± 0.48	
HM3		1.20 ± 0.40		2.21 ± 0.50		2.32 ± 0.84	
HW	APTT, s	42.2 ± 8.86	0.02 **	50.7 ± 10.2	0.53	60.9 ±12.9	0.01 *
HMII		41.3 ± 9.19		56.7 ± 19.1		49.9 ± 6.27	
HM3		37.7 ± 6.62		53.1 ± 15.7		50.7 ± 12.5	
HW	LDH, U/L	360.2 ± 210.3	0.15	234.8 ± 105.6	0.002 **	271.1 ± 118.4	0.02 **
HMII		330.5 ± 178.8		351.5 ± 92.1		356.8 ± 181.3	
HM3		248.1 ± 110.7		251.1 ± 88.0		213.8 ± 55.2	
HW	Creatinine, mg/dL	0.97 ± 0.45	0.75	0.78 ± 0.40	0.04 **	1.18 ± 0.51	0.84
HMII		1.13 ± 0.44		0.95 ± 0.53		1.03 ± 0.31	
HM3		3.05 ± 14.0		1.02 ± 0.21		1.08 ± 0.28	
HW	CRP, mg/dL	1.95 ± 3.01	0.62	0.11 ± 0.07	0.05 **	2.08 ± 2.90	0.58
HMII		1.64 ± 2.16		0.73 ± 1.06		1.14 ± 1.72	
HM3		1.19 ± 2.07		1.09 ± 1.58		0.66 ± 0.78	
HW	LVEF, %	21.3 ± 5.01	0.36	23.2 ± 4.82	0.02 *	24.7 ± 4.04	0.74
HMII		23.1 ± 6.41		31.0 ± 6.69		28.5 ± 7.46	
HM3		21.4 ± 4.67		25.1 ± 4.32		25.5 ± 3.70	

Continuous variables are presented as mean ± SD. Significant *p*-value with one asterisk (*) from one-way ANOVA; significant *p*-value with double asterisks (**) from Kruskal–Wallis test; the significant *p* value (*p* < 0.05) is labeled in bold; HW, HeartWare HVAD; HMII, HeartMate II; HM3, HeartMate 3; APTT, activated partial thromboplastin time; LDH, lactate dehydrogenase; CRP, C—reactive protein; LVEF, left ventricular ejection fraction.

**Table 3:** Multiple linear regression analysis to identify the influence of independent factors on the TEG CI level.

Characteristics	Unstandardized Coefficients		Standardized Coefficients	t	*p* Value	95% Confidence Interval	
	B	Std.Error	Beta			Lower Bound	Upper Bound
(Constant)	2.01	3.56		0.56	0.58	−5.21	9.22
Age	−0.001	0.03	−0.005	−0.04	0.97	−0.07	0.07
BMI	0.03	0.08	0.06	0.38	0.70	−0.12	0.18
Device type	0.57	0.46	0.18	1.23	0.23	−0.37	1.51
Warfarin dose	−1.03	0.31	−0.49	−3.33	0.002 *	−1.66	−0.40
Aspirin dose	0.02	0.01	0.27	1.86	0.07	−0.002	0.05
*VKORC1*, rs8050894	0.79	1.02	0.15	0.78	0.44	−1.27	2.86
*VKORC1*, rs9923231	−3.25	1.49	−0.47	−2.17	0.04 *	−6.28	−0.22
*ITGB3*, rs5918	2.18	1.08	0.41	2.03	0.05 *	0.003	4.36
*UGT1A6*, rs2070959	−0.55	0.72	−0.10	−0.76	0.45	−2.02	0.92

The significant *p*-value (*p* < 0.05) is labeled in bold with an asterisk (*); BMI, body mass index.

**Table 4:** Multinomial logistic regression analysis for the identification of the influence of independent factors between LVAD types.

Device Type	Characteristics	B	Std.Error	*p* Value	Odds Ratio	95% Confidence Interval	
						Lower Bound	Upper Bound
HW	Intercept	3.63	2.52	0.15			
	Age	0.02	0.03	0.40	1.02	0.97	1.08
	BMI	−0.23	0.09	0.01 *	0.79	0.66	0.95
	Warfarin dose, mg/day	0.19	0.27	0.46	1.22	0.72	2.07
	*VKORC1* rs9923231, CC genotype	−1.66	1.22	0.17	0.19	0.02	2.07
	*VKORC1* rs9923231, CT/TT genotype	0 ^b^					
	*ITGB3* rs5918, TT genotype	−0.46	0.62	0.46	0.63	0.19	2.14
	*ITGB3* rs5918, TC/CC	0 ^b^					
HMII	Intercept	−3.30	2.25	0.14			
	Age	0.006	0.02	0.81	1.01	0.96	1.05
	BMI	0.14	0.06	0.02 *	1.15	1.02	1.29
	Warfarin dose, mg/day	−0.23	0.23	0.32	0.79	0.50	1.25
	*VKORC1* rs9923231, CC genotype	0.24	0.78	0.76	1.27	0.27	5.86
	*VKORC1* rs9923231, CT/TT genotype	0 ^b^					
	*ITGB3* rs5918, TT genotype	−1.09	0.53	0.04 *	0.33	0.12	0.94
	*ITGB3* rs5918, TC/CC genotype	0 ^b^					

The significant *p*-value (*p* < 0.05) is labeled in bold with an asterisk (*); HW, HeartWare HVAD; HMII, HeartMate II; BMI, body mass index. b, this parameter is set to zero because it is redundant.

## Data Availability

National Laboratory Astana (contact via phone or mail) for researchers who meet the criteria for access to confidential data. The data underlying the results presented in the study are available from the authors, phone number: +7-7172706501, mail: akilzhanova@nu.edu.kz.

## Supplementary Materials

The following supporting information can be downloaded at: https://www.mdpi.com/article/10.3390/jcm12237235/s1, Table S1: Comparative analysis of biochemical parameters between HeartWare HVAD, HeartMate II and HeartMate 3; Table S2: Comparative analysis of thromboelastography parameters between HeartWare HVAD, HeartMate II and HeartMate 3; Table S3: Comparative analysis of thromboelastography parameters between HF patients without complications, with thrombosis and bleeding complications; Table S4: List of SNP characteristics; Table S5: The distributions of allelic and genotype frequencies of 21 SNPs between Control group and HF patients; Table S6: The distributions of allelic and genotype frequencies of 21 SNPs between HF patients with/without complications; Table S7: Association analysis of SNPs for LVAD complications in HF patients; Table S8: Pearson Correlation analysis; Table S9: The distributions of allelic and genotype frequencies of *ITGB3* gene polymorphism rs5918 in the group of HF patients with complications (*n*=24); Table S10. The distributions of allelic and genotype frequencies of 21 SNPs between HeartWare HVAD, HeartMate II and HeartMate 3.

## References

[1] Berardi C., Bravo C.A., Li S., Khorsandi M., Keenan J.E., Auld J., Rockom S., Beckman J.A., Mahr C. (2022). The History of Durable Left Ventricular Assist Devices and Comparison of Outcomes: HeartWare, HeartMate II, HeartMate 3, and the Future of Mechanical Circulatory Support. J. Clin. Med..

[2] Garbade J., Gustafsson F., Shaw S., Lavee J., Saeed D., Pya Y., Krabatsch T., Schmitto J.D., Morshuis M., Chuang J. (2019). Postmarket Experience With HeartMate 3 Left Ventricular Assist Device: 30-Day Outcomes From the ELEVATE Registry. Ann. Thorac. Surg..

[3] Kadakia S., Moore R., Ambur V., Toyoda Y. (2016). Current status of the implantable LVAD. Gen. Thorac. Cardiovasc. Surg..

[4] Austin M.A., Maynes E.J., Gadda M.N., O’Malley T.J., Morris R.J., Shah M.K., Pirlamarla P.R., Alvarez R.J., Entwistle J.W., Massey H.T. (2021). Continuous-flow LVAD exchange to a different pump model: Systematic review and meta-analysis of the outcomes. Artif. Organs.

[5] Eckman P.M., John R. (2012). Bleeding and thrombosis in patients with continuous-flow ventricular assist devices. Circulation.

[6] Kirklin J.K., Naftel D.C., Kormos R.L., Pagani F.D., Myers S.L., Stevenson L.W., Acker M.A., Goldstein D.L., Silvestry S.C., Milano C.A. (2014). Interagency registry for mechanically assisted circulatory support (INTERMACS) analysis of pump thrombosis in the HeartMate II left ventricular assist device. J. Hear. Lung Transplant..

[7] Kormos R.L., Cowger J., Pagani F.D., Teuteberg J.J., Goldstein D.J., Jacobs J.P., Higgins R.S., Stevenson L.W., Stehlik J., Atluri P. (2019). The Society of Thoracic Surgeons Intermacs database annual report: Evolving indications, outcomes, and scientific partnerships. J. Hear. Lung Transplant..

[8] Bravo C.A., Fried J.A., Willey J.Z., Javaid A., Mondellini G.M., Braghieri L., Lumish H., Topkara V.K., Kaku Y., Witer L. (2021). Presence of Intracardiac Thrombus at the Time of Left Ventricular Assist Device Implantation Is Associated With an Increased Risk of Stroke and Death. J. Card. Fail..

[9] Mehra M.R., Uriel N., Naka Y., Cleveland J.C., Yuzefpolskaya M., Salerno C.T., Walsh M.N., Milano C.A., Patel C.B., Hutchins S.W. (2019). A fully magnetically levitated left ventricular assist device—Final report. N. Engl. J. Med..

[10] Wever-Pinzon O., Naka Y., Garan A.R., Takeda K., Pan S., Takayama H., Mancini D.M., Colombo P., Topkara V.K. (2016). National trends and outcomes in device-related thromboembolic complications and malfunction among heart transplant candidates supported with continuous-flow left ventricular assist devices in the United States. J. Hear. Lung Transplant..

[11] Zhalbinova M.R., Rakhimova S.E., Kozhamkulov U.A., Akilzhanova G.A., Kaussova G.K., Akilzhanov K.R., Pya Y.V., Lee J.H., Bekbossynova M.S., Akilzhanova A.R. (2022). Association of genetic polymorphisms with complications of implanted LVAD devices in patients with congestive heart failure: A Kazakhstani study. J. Pers. Med..

[12] Molina E.J., Shah P., Kiernan M.S., Cornwell W.K., Copeland H., Takeda K., Fernandez F.G., Badhwar V., Habib R.H., Jacobs J.P. (2021). The Society of Thoracic Surgeons Intermacs 2020 Annual Report. Ann. Thorac. Surg..

[13] Topkara V.K., Knotts R.J., Jennings D.L., Garan A.R., Levin A.P., Breskin A., Castagna F., Cagliostro B., Yuzefpolskaya M., Takeda K. (2016). Effect of CYP2C9 and VKORC1 gene variants on warfarin response in patients with continuous-flow left ventricular assist devices. ASAIO J..

[14] Starling R.C., Moazami N., Silvestry S.C., Ewald G., Rogers J.G., Milano C.A., Rame J.E., Acker M.A., Blackstone E.H., Ehrlinger J. (2014). Unexpected abrupt increase in left ventricular assist device thrombosis. N. Engl. J. Med..

[15] Topkara V.K., O’Neill J.K., Carlisle A., Novak E., Silvestry S.C., Ewald G.A. (2014). HeartWare and HeartMate II Left ventricular assist devices as bridge to transplantation: A comparative analysis. Ann. Thorac. Surg..

[16] Mehra M.R., Goldstein D.J., Uriel N., Cleveland J.C., Yuzefpolskaya M., Salerno C., Walsh M.N., Milano C.A., Patel C.B., Ewald G.A. (2018). Two-Year outcomes with a magnetically levitated cardiac pump in heart failure. N. Engl. J. Med..

[17] Rogers J.G., Pagani F.D., Tatooles A.J., Bhat G., Slaughter M.S., Birks E.J., Boyce S.W., Najjar S.S., Jeevanandam V., Anderson A.S. (2017). Intrapericardial left ventricular assist device for advanced heart failure. N. Engl. J. Med..

[18] Cheng A., Williamitis C.A., Slaughter M.S. (2014). Comparison of continuous-flow and pulsatile-flow left ventricular assist devices: Is there an advantage to pulsatility?. Ann. Cardiothorac. Surg..

[19] Chen Z., Koenig S.C., Slaughter M.S., Griffith B.P., Wu Z.J. (2018). Quantitative Characterization of Shear-Induced Platelet Receptor Shedding: Glycoprotein Ibα, Glycoprotein VI, and Glycoprotein IIb/IIIa. ASAIO J..

[20] Chen Z., Zhang J., Kareem K., Tran D., Conway R.G., Arias K., Griffith B.P., Wu Z.J. (2019). Device-induced platelet dysfunction in mechanically assisted circulation increases the risks of thrombosis and bleeding. Artif. Organs.

[21] Slaughter M.S., Rogers J.G., Milano C.A., Russell S.D., Conte J.V., Feldman D., Sun B., Tatooles A.J., Delgado R.M., Long J.W. (2009). Advanced heart failure treated with continuous-flow left ventricular assist device. N. Engl. J. Med..

[22] Chen Y., Kuehl G.E., Bigler J., Rimorin C.F., Schwarz Y., Shen D.D., Lampe J.W. (2007). UGT1A6 polymorphism and salicylic acid glucuronidation following aspirin. Pharmacogenetics Genom..

[23] Xia R., Varnado S., Graviss E.A., Nguyen D.T., Cruz-Solbes A., Guha A., Krisl J.C. (2020). Role of thromboelastography in predicting and defining pump thrombosis in left ventricular assist device patients. Thromb. Res..

[24] Volod O., Lam L.D., Lin G., Kam C., Kolyouthapong K., Mac J., Mirocha J., Ambrose P.J., Czer L.S.C., Arabia F.A. (2017). Role of thromboelastography platelet mapping and international normalized ratio in defining “normocoagulability” during anticoagulation for mechanical circulatory support devices: A pilot retrospective study. ASAIO J..

[25] Boyle A.J., Jorde U.P., Sun B., Park S.J., Milano C.A., Frazier O.H., Sundareswaran K.S., Farrar D.J., Russell S.D. (2014). Pre-Operative risk factors of bleeding and stroke during left ventricular assist device support. J. Am. Coll. Cardiol..

[26] Piche S.L., Nei S.D., Frazee E., Schettle S.D., Boilson B.A., Plevak M.F., Dierkhising R.A., Stulak J.M. (2019). Baseline thromboelastogram as a predictor of left ventricular assist device thrombosis. ASAIO J..

[27] Zhang J.-H., Tang X.-F., Zhang Y., Wang J., Yao Y., Ma Y.-L., Xu B., Gao R.-L., Gao Z., Chen J. (2014). Relationship between ABCB1 polymorphisms, thromboelastography and risk of bleeding events in clopidogrel-treated patients with ST-elevation myocardial infarction. Thromb. Res..

[28] Franchi F., Hammad J.S., Rollini F., Tello-Montoliu A., Patel R., Darlington A., Kraemer D.F., Cho J.R., DeGroat C., Bhatti M. (2015). Role of thromboelastography and rapid thromboelastography to assess the pharmacodynamic effects of vitamin K antagonists. J. Thromb. Thrombolysis.

[29] Dunham C.M., Rabel C., Hileman B.M., Schiraldi J., Chance E.A., Shima M.T., Molinar A.A., Hoffman D.A. (2014). TEG^®^ and RapidTEG^®^ are unreliable for detecting warfarin-coagulopathy: A prospective cohort study. Thromb. J..

[30] Awad M., Czer L.S.C., Soliman C., Mirocha J., Ruzza A., Pinzas J., Rihbany K., Chang D., Moriguchi J., Ramzy D. (2015). Prevalence of Warfarin Genotype Polymorphisms in Patients with Mechanical Circulatory Support. ASAIO J..

[31] Hu J., Mondal N.K., Sorensen E.N., Cai L., Fang H.-B., Griffith B.P., Wu Z.J. (2014). Platelet glycoprotein Ibα ectodomain shedding and non-surgical bleeding in heart failure patients supported by continuous-flow left ventricular assist devices. J. Heart Lung Transplant..

[32] Chen Z., Mondal N.K., Ding J., Koenig S.C., Slaughter M.S., Griffith B.P., Wu Z.J. (2015). Activation and shedding of platelet glycoprotein IIb/IIIa under non-physiological shear stress. Mol. Cell. Biochem..

[33] Grinshtein Y.I., Kosinova A.A., Grinshtein I.Y., Subbotina T.N., Savchenko A.A. (2018). The Prognostic Value of Combinations of Genetic Polymorphisms in the ITGB3, ITGA2, and CYP2C19*2 Genes in Predicting Cardiovascular Outcomes After Coronary Bypass Grafting. Genet. Test. Mol. Biomark..

[34] Kucharska-Newton A.M., Monda K.L., Campbell S., Bradshaw P.T., Wagenknecht L.E., Boerwinkle E., Wasserman B.A., Heiss G. (2011). Association of the platelet GPIIb/IIIa polymorphism with atherosclerotic plaque morphology: The Atherosclerosis Risk in Communities (ARIC) Study. Atherosclerosis.

[35] Potapov E.V., Ignatenko S., Nasseri B.A., Loebe M., Harke C., Bettmann M., Doller A., Regitz-Zagrosek V., Hetzer R. (2004). Clinical significance of PlA polymorphism of platelet GP IIb/IIIa receptors during long-term VAD support. Ann. Thorac. Surg..

